# Analysis of Single Nucleotide Polymorphisms of *STK32B, PPARGC1A* and *CTNNA3* Gene With Sporadic Parkinson's Disease Susceptibility in Chinese Han Population

**DOI:** 10.3389/fneur.2018.00387

**Published:** 2018-05-30

**Authors:** Chang-he Shi, Yuan Cheng, Mi-bo Tang, Yu-tao Liu, Zhi-hua Yang, Fang Li, Yu Fan, Jing Yang, Yu-ming Xu

**Affiliations:** ^1^Department of Neurology, The First Affiliated Hospital of Zhengzhou University, Zhengzhou, China; ^2^Institute of Clinical Medicine, The First Affiliated Hospital of Zhengzhou University, Zhengzhou, China

**Keywords:** single nucleotide polymorphisms (SNPs), Parkinson's disease, *STK32B*, *PPARGC1*, *CTNNA3*

## Abstract

Recently, five novel single nucleotide polymorphisms (SNPs), rs10937625 in *STK32B* (serine/threonine kinase 32B), rs17590046 in *PPARGC1A* (peroxisome proliferator-activated receptor gamma coactivator 1-alpha), and rs12764057, rs10822974, and rs7903491 in *CTNNA3* (catenin alpha 3), were found to be associated with increased risk of essential tremor (ET) in a genome-wide association study (GWAS)in individuals of Caucasian ancestry. Considering the overlap between ET and Parkinson's disease (PD) in pathological features and clinical manifestations, a case-control study comprising 546 PD patients and 550 control subjects was carried out to examine whether the same variants were also associated with PD in Chinese Han population. However, the above variants did not show an association with PD. Our results suggested that these variants do not play a major role in PD in the Chinese population, Actually, the clinical overlap between PD and ET is under debate. In our Chinese Han cohort, we did not verify potential genetic pleiotropy between two diseases, which may indicated that etiology and pathobiology of PD and ET are distinct. Thus, a more comprehensive study such as a multi-center study may be helpful to evaluate the relationship between the five new susceptible loci and PD in Chinese Han population in the future.

## Introduction

Parkinson's disease (PD) is the second most common neurodegenerative disorder, and affects approximately 1.7 million individuals (aged ≥ 65 years) in China (1). Although the exact cause of PD remains unknown, accumulating evidence has demonstrated that genetic background is a potential contributor to the pathogenesis of this disease (2, 3).

Genome-wide association study (GWAS) is a powerful tool for genetic association studies, many GWAS-related polymorphic loci, such as single nucleotide polymorphisms (SNPs) in *SNCA, GBA*, and *LRRK2* have been reported to be associated with the risk of PD (4–8). Recently, a GWAS reported that a progress has been made in research on gene polymorphism in essential tremor (ET). Five new loci (rs10937625 in *STK32B* (serine/threonine kinase 32B), rs17590046 in *PPARGC1A* [peroxisome proliferator-activated receptor gamma coactivator 1-alpha), and rs12764057, rs10822974, and rs7903491 in *CTNNA3* (catenin alpha 3)] have been identified, and are significantly associated with increased risk of ET in individuals of Caucasian ancestry (9). Actually, the clinical overlap between PD and ET is under debate. Although the underline mechanisms between these two disorders are distinct (10), the overlapping clinical features suggest that PD and ET may share common risk causes such as genetic factor. The risk to develop PD after an initial diagnosis of ET was four-fold when compared to a non-ET population (11), and individuals with a PD-diagnosed relative are more likely to develop ET (12). The GWAS *LINGO1* variant has been implicated in etiologic links between ET and PD (13). Moreover, there are several variants associated with PD have also been observed in ET patients (14–16). Thus, it would be meaningful to explore whether the ET-related genetic variants are also associated with PD.

Consequently, a case-control study comprising 546 PD patients and 550 healthy controls was performed to investigate the association between the five new loci and PD in Chinese Han population. In fact, similar studies have been published in two independent groups (17, 18), and one was performed in China (18). However, there is still a lack of study in northern China. As far as we know, this research is the first study to detect the relationship between the three new loci and the risk of PD in North Chinese population.

## Methods

As shown in Table 1, 546 mainland Chinese with sporadic PD and 550 age- and sex- matched healthy individuals were recruited from The First Affiliated Hospital of Zhengzhou University. PD patients with onset age younger than 50 years old were classified into early-onset PD (EOPD) and those with onset age older than or equal to 50 years were late-onset PD (LOPD). There are 98 EOPD patients (age < 50) and 448 LOPD patients (age ≥ 50), 248 patients with tremor dominant PD (TD) and 298 patients with indeterminate type or pace and instable gate PD (PIGD). This classification is determined by the ratio of tremor score to postural instability and mean gait disorder scores (TD/PIGD), and the scores of motor symptoms can be determined by the Unified Parkinson's Disease Rating Scale (UPDRS). TD is categoried with the criterion that TD total score/PIGD total score ≧ 1.5, and when TD total score/PIGD total score ≦ 1, it is categoried as PIGD, similar to previously published method (19, 20). All patients underwent a standardized neurological examination by two movement disorder specialists, and the diagnosis was made according to the criteria of the United Kingdom PD Society Brain Bank (21). The exclusion criteria include: a variety of secondary Parkinson's syndrome (including traumatic, neoplastic, drug-induced, toxic, vascular, hydrocephalus, etc.), and Parkinson's-plus syndrome; primary Parkinson's disease with surgery or gamma knife Treatment; schizophrenia, or other patients with severe mental illness; severe heart, liver, kidney, and other organ damage should also be ruled out. All subjects were ethnic Hans and gave informed consent. The study received approval from the institutional ethics committee. Genomic DNA was extracted from peripheral blood lymphocytes using standard procedures (22). We designed specific primers for each single nucleotide polymorphism (SNP) site, and polymerase chain reaction analysis was conducted. The detailed primer sequences are shown in Table 4. Both cases and controls were genotyped by Sanger sequencing. DNASTAR Lasergene MegAlign (v7.1.0) and Chromas (v2.33) were used to conduct sequence alignment. PASS (v11.0) was used to calculate statistical power. The Hardy-Weinberg equilibrium for genotype-frequency control subjects was examined. Chi-squared tests were adopted to compare differences of allele frequency, and Logistic regression analysis was applied to evaluate the association of SNP with the risk of PD. Bonferroni correction was used to adjust for multiple testing, and we set the significance at 0.01.

**Table 1:** Baseline Characteristics.

**Characteristics**	**Cases = 546**		**Control** = **550**	
**GENDER**				
Male (*n*, %)	317	58.1%	308	56%
Female (*n*, %)	229	41.9%	242	44%
Age at collection	61.48 ± 10.611		62.39 ± 8.39	

*There were no significant statistical differences in age and gender ratio between case and control. The T value and final P-value are −1.577 and 0.115 respectively, and the Chi-square value and P value are 0.474 and 0.491 respectively*.

## Results

Clinical characteristics of the subjects are shown in Table 1. The characteristics of the SNPs are shown in Table 2. All selected SNPs were at Hardy-Weinberg equilibrium (*P* > 0.05) in the control group (Table 2).

**Table 2:** Characteristics of included SNPs.

**SNP**	**Chr**	**Gene**	**Alleles**	**MAF (1,000g-CHBS)**	**MAF (observed)**	**Ancestral Allele**	**Test for HWE (*P*-Value)**	**Crude *P*-value (allele)**	**Adjusted *P*-value (allele)**	**OR**	**95% CI**
rs10937625	4	STK32B	C/T	17% (C)	17.9% (C)	T	0.204	0.36	0.39	0.903	(0.726,1.124)
rs17590046	4	PPARGC1A	C/T	6.3% (C)	9.5% (C)	T	0.073	0.132	0.152	0.802	(0.601, 1.069)
rs12764057	10	CTNNA3	T/G	27.2% (G)	32.3% (G)	T	0.056	0.51	0.54	1.062	(0.888, 1.27)
rs10822974	10	CTNNA3	A/G	47.1% (A)	42.3% (A)	A	0.380	0.749	0.782	0.973	(0.821, 1.152)
rs7903491	10	CTNNA3	A/G	43.2% (G)	43.1% (G)	A	0.535	0.622	0.652	0.958	(0.809, 1.135)

*Chr, chromosome; CI, confidence interval; OR, odds ratio; SNP, single-nucleotide polymorphism; MAF, Minor Allele Frequency; 1,000g-CHB,1,000g-Chinese Han Beijing*.

In current study, no associations were detected between rs12764057 (genotype *p* = 0.065; allele *p* = 0.54), rs10822974 (genotype *p* = 0.811; allele *p* = 0.782), rs10937625 (genotype *p* = 0.677; allele *p* = 0.39), rs7903491 (genotype *p* = 0.682; allele *p* = 0.652), rs17590046 (genotype *p* = 0.436; allele *p* = 0.152) and PD in our Chinese Han cohort (Table 3). In an analysis of additive, dominant, and recessive genetic models, none of the five polymorphic variants showed evidence of an association with PD in our samples (Table 3). Additionally, we found no associations between TD and PIGD when stratified in terms of clinical subtypes (Table S1). After gender and age stratification, yet no associations were detected for subgroups of males or females and EOPD or LOPD between patients and controls. (Tables S2, S3).

**Table 3:** Genotype distributions of included SNPs and single-nucleotide polymorphism associated with PD according to different genetic models.

**SNP**		**Genotype**			**Crude *P* (genotype)**	**Adjusted *P* (genotype)**	**Model**	**Crude *P* (model)**	**Adjusted *P* (model)**	**OR 95% CI**
							
rs10937625		T/T = 2	C/T = 1	C/C = 3	0.666	0.677	Addition	0.405	0.413	0.701 (0.300,1.641)
	Case	362 (66.3%)	164 (30%)	20 (3.7%)			Dominant	0.728	0.737	0.951 (0.708, 1.276)
	Control	373 (67.8%)	165 (30%)	12 (2.2%)			Recessive	0.372	0.382	0.692 (0.304,1.578)
rs17590046		T/T = 1	C/T = 2	C/C = 3	0.459	0.436	Addition	0.271	0.247	1.274 (0.846, 1.919)
	Case	455 (83.3%)	89 (16.5%)	2 (0.2%)			Dominant	0.238	0.217	0.775 (0.517, 1.162)
	Control	438 (79.6%)	110 (20%)	2 (0.4%)			Recessive	0.556	0.571	0.499 (0.045, 5.528)
rs12764057		T/T = 2	G/T = 1	G/G = 3	0.064	0.065	Addition	0.177	0.185	0.701 (0.415,1.186)
	Case	250 (45.8%)	246 (45%)	50 (9.2%)			Dominant	0.055	0.054	1.315 (0.996, 1.738)
	Control	237 (43.1%)	263 (47.8%)	50 (9.1%)			Recessive	0.450	0.468	0.830 (0.502, 1.373)
rs10822974		G/G = 1	G/A = 2	A/A = 3	0.793	0.811	Addition	0.537	0.549	1.104 (0.807, 1.511)
	Case	172 (31.5%)	282 (51.7%)	92 (16.8%)			Dominant	0.773	0.806	0.955 (0.662, 1.378)
	Control	180 (32.7%)	278 (50.6%)	92 (16.7%)			Recessive	0.619	0.622	1.078 (0.800, 1.454)
rs7903491		G/G = 2	G/A = 1	A/A = 3			Addition	0.376	0.383	0.845 (0.597, 1.233)
	Case	108 (19.8%)	260 (47.6%)	178 (32.6%)	0.675	0.682	Dominant	0.965	0.964	1.007 (0.749, 1.353)
	Control	96 (17.5%)	276 (50.2%)	178 (32.3%)			Recessive	0.412	0.419	0.863 (0.604, 1.233)

*CI, confidence interval; OR, odds ratio; SNP, single-nucleotide polymorphism; model, Genetic model*.

**Table 4:** The detailed primer sequences.

**SNP**	**Gene**	**Primer**	
		**Forward**	**Reverse**
rs10937625	STK32B	GTACTGGAGCTACAGAGAAGGC	AGGCAGCACTAGACAGTGAGAC
rs17590046	PPARGC1A	AGTGCCTGATTCTGCCTGAGAG	CTGGAGCAAGTGGTCACCTGAA
rs12764057	CTNNA3	CAGTTCCCTGCCAATCTTTCCA	TCTGTATGCATCTGCCAGGTTG
rs10822974	CTNNA3	AGGCAACCACTTAGGCAACTCA	GCTTGGGTCCCTGGCAAAGT
rs7903491	CTNNA3	AGGTGCTCTGATGTTGGGTACA	GGCTACATGCGCGGCTAATTG

*SNP, single-nucleotide polymorphism*.

## Discussion

GWAS is a powerful tool for genetic association studies; however, dependent replication is still an important step in distinguishing between true- and false-positive genetic correlations (23). Recently, a study in Singapore verified a previously reported GWAS-linked ET risk locus, *PPARGC1A* (rs17590046), in Asian patients (9, 24). The pathological features and clinical manifestations of ET and PD overlap. Thus, it is meaningful to verify GWAS-identified ET risk loci in other PD populations. Our case-control study found no association between the five novel SNPs and PD in Chinese Han population, which is consistent with the previous studies (17, 18). It is worth mentioning that there are differences in genes between southern and northern Chinese population (25). Our cohort is mainly from the northern regions of China, together with Zhang Y's research which object is the Chinese population of southern area (18), provide more comprehensive data on the Chinese population.

To date, a series of studies were performed to identify potential genetic factors which may contribute to both PD and ET. However, the results are frustrating and confusing. It was reported rs9652490 in *LINGO1* increased the risk of ET from European and American populations in a GWAS (26). Another research presented that rs9652490 is associated with both ET and PD of Caucasian origin from North America (13). However, a recent research showed no association between rs9652490 and PD in Chinese Han population (27). Another SNP *SLC1A2* rs3794087 was associated with ET in a GWAS from Europeans (26), but the result of related study about ET and PD in Chinese Han population is controversial (28–30). All these publications indicated genetic heterogeneity of ET and PD may exist in various populations, which may largely account for the negative results in this study.

*STK32B* and *CTNNA3* are two protein-coding genes, but the current study has not found any association with pathogenic pathways of Parkinson's disease. However, It is worth mentioning that among the 3 genes described above, *PPARGC1A* plays an important role in the development and progression of neurodegenerative diseases such as Amyotrophic lateral sclerosis and Huntington's disease (31, 32). As we all known, mitochondrial dysfunction and oxidative stress have been strongly linked to PD (33, 34). PGC-1a (an alias for *PPARGC1A*), which regulates mitochondrial biogenesis and expression of many antioxidant genes, plays an important role in protection against 1-methyl-4-phenyl-1,2,3,6-tetrahydropyridine (MPTP) toxicity (35), and expression of genes regulated by PGC-1a are reduced in substantia nigra neurons at early stages of PD (36). Polymorphism research has shown possible associations between PGC-1α SNPs rs6821591 and rs2970848 and age of PD onset in individuals of Caucasian ancestry (37). All of these suggest that *PPARGC1A* may be involved in the pathogenesis of PD. Accordingly, follow-up studies in Chinese Han population should focus more on *PPARGC1A*The observed minor allele frequencies of the 5 SNPs were similar to those reported in the 1,000 Genomes Project in Chinese Han population (Table 2). However, our study also had several limitations. First, there might be potential selection bias due to the hospital-based study design. Second, the statistical power value of each site does not exceed 80% because of the limited sample size (the statistical power value of rs10937625, rs17590046, rs12764057, rs10822974, rs7903491 are 12.4, 5.8, 34.7, 9.5, 17.7% respectively), thus some positive results might not be detected. Large-sample, multi-center trials are required to better comprehend the contribution of these 5 SNPs to PD susceptibility. Third, although all the PD patients we enrolled have no family history, some patients are still likely to carry other pathogenic genes such as *Parkin* and *PINK1*, which may have some impacts on our results.

In conclusion, our results confirmed that the five novel variants are not associated with sporadic PD in Chinese Han population. Additional studies are needed to elucidate the precise mechanism of the association between PD and these variants.

## Ethics statement

Commitments (applicants):

1. This study doesn't intervene the whole process of diagnosis and treatment, and doesn't affect the patients medication such as diagnosis and treatment
2. Informed consent of subjects was obtained from all the subjects, and explain the purpose and significance of research to them
3. We will never give patient information to others, anonymously to the statistics, and the research will bring benefits to diagnosis and treatment, but the subjects will not take on additional risk.

## Author contributions

JY, YC, CS, and YX: The conception or design of the work; JY, YC, MT, ZY, FL, YF, CS, and YX: Drafting the work or revising it; JY, YC, MT, ZY, FL, YF, CS, and YX: Final approval of the version to be published; JY, YC, MT, ZY, FL, YF, CS, and YX: Agreement to be accountable for all aspects of the work.

## Conflict of interest statement

The authors declare that the research was conducted in the absence of any commercial or financial relationships that could be construed as a potential conflict of interest.

## Supplementary material

Table S1 Genotype distributions of included SNPs and single-nucleotide polymorphism associated with PD according to different clinical subtypes.

Table S2 Genotype distributions of included SNPs and single-nucleotide polymorphism associated with PD according to different gender.

Table S3 Genotype distributions of included SNPs and single-nucleotide polymorphism associated with PD according to different onset age.
