# Supplementary material for: Analysis of Single Nucleotide Polymorphisms of STK32B, PPARGC1A and CTNNA3 Gene With Sporadic Parkinson's Disease Susceptibility in Chinese Han Population
Source: Front Neurol. 2018 May 30;9:387. doi: 10.3389/fneur.2018.00387 (PMC5989317; doi:10.3389/fneur.2018.00387)
Supplement: Table S1 — Genotype distributions of included SNPs and single-nucleotide polymorphism associated with PD according to different clinical subtypes. [file Table_1.DOCX]

Supplementary Material

### Analysis of single nucleotide polymorphisms of *STK32B*, *PPARGC1A* and *CTNNA3* Gene with Sporadic Parkinson’s disease Susceptibility in Chinese Han population

Chang-he Shi, MD, PhD^1, #^, Yuan Cheng, MD^1, 2 #^, Mi-bo Tang, MD^1, 2^, Yu-tao Liu, MD, PhD ^1^, Zhi-hua Yang, MD^1, 2^, Fang Li, MD^1, 2^, Yu Fan, MD^1, 2^, Jing Yang, MD, PhD ^1 *^, Yu-ming Xu, MD, PhD^1, *^

1 Department of Neurology, The First Affiliated Hospital of Zhengzhou University, Zhengzhou, 450052, Henan, China.
2 Institute of Clinical Medicine, The First Affiliated Hospital of Zhengzhou University, Zhengzhou, 450052, Henan, China.

#: These authors contributed equally to this article
*: Corresponding Author: Yu-ming Xu and Jing Yang
E-mail: xuyuming@zzu.edu.cn; yangjing9527@126.com

| SNP clinical type | | genotype | | | *P*（genotype） | *P*（Alleles） | OR(95%CI) (Alleles) |
| --- | --- | --- | --- | --- | --- | --- | --- |
| rs10937625 |  | T/T(362) | C/T(164) | C/C(20) | 0.739 | 0.834 | 0.968(0.713,1.314) |
|  | TD (248) | 162 | 78 | 8 |  |  |  |
|  | PIGD (298) | 200 | 86 | 12 |  |  |  |
| rs17590046 |  | T/T(455) | C/T(89) | C/C(2) | 0.94 | 0.787 | 1.061(0.692,1.627) |
|  | TD (248) | 208 | 39 | 1 |  |  |  |
|  | PIGD (298) | 247 | 50 | 1 |  |  |  |
| rs12764057 |  | T/T(250) | G/T(246) | G/G(50) | 0.843 | 0.587 | 1.074(0.831,1.387) |
|  | TD (248) | 116 | 111 | 21 |  |  |  |
|  | PIGD (298) | 134 | 135 | 29 |  |  |  |
| rs10822974 |  | G/G(172) | G/A(282) | A/A(92) | 0.511 | 0.487 | 1.089(0.856,1.386) |
|  | TD (248) | 84 | 122 | 42 |  |  |  |
|  | PIGD (298) | 88 | 160 | 50 |  |  |  |
| rs7903491 |  | G/G(108) | G/A(260) | A/A(178) | 0.815 | 0.145 | 1.199(0.939,1.531) |
|  | TD (248) | 52 | 116 | 80 |  |  |  |
|  | PIGD (298) | 56 | 144 | 98 |  |  |  |

**Table S1**

| SNP gender | | | genotype | | | *P*（genotype） | *P*（Alleles） | OR(95%CI) (Alleles) |
| --- | --- | --- | --- | --- | --- | --- | --- | --- |
|  |  |  |  |  |  |  |  |  |
| rs10937625 |  | | T/T (735) | C/T (329) | C/C (32) |  | | |
|  | **male** | case (317) | 213 | 95 | 9 | 0.996 | 0.928 | 1.013 (0.759, 1.353) |
|  |  | control (308) | 206 | 93 | 9 |  |  |  |
|  | **female** | case (229) | 153 | 69 | 7 | 0.989 | 0.888 | 0.976 (0.700, 1.362) |
|  |  | control (242) | 163 | 72 | 7 |  |  |  |
| rs17590046 |  | | T/T (893) | C/T (199) | C/C (4) |  | | |
|  | **male** | case (317) | 259 | 57 | 1 | 0.998 | 0.947 | 1.013 (0.692, 1.482) |
|  |  | control (308) | 251 | 56 | 1 |  |  |  |
|  | **female** | case (229) | 186 | 42 | 1 | 0.998 | 0.957 | 0.988 (0.640, 1.526) |
|  |  | control (242) | 197 | 44 | 1 |  |  |  |
| rs12764057 |  | | T/T (487) | G/T (509) | G/G (100) |  | | |
|  | **male** | case (317) | 138 | 147 | 32 | 0.913 | 0.76 | 0.964 (0.761, 1.220) |
|  |  | control (308) | 136 | 144 | 28 |  |  |  |
|  | **female** | case (229) | 102 | 106 | 21 | 0.875 | 0.713 | 0.95 (0.722, 1.25) |
|  |  | control (242) | 110 | 113 | 19 |  |  |  |
| rs10822974 |  | | G/G (352) | G/A (560) | A/A (184) |  | | |
|  | **male** | case (317) | 112 | 162 | 43 | 0.449 | 0.27 | 1.136 (0.906, 1.424) |
|  |  | control (308) | 99 | 157 | 52 |  |  |  |
|  | **female** | case (229) | 72 | 118 | 39 | 0.557 | 0.311 | 1.142 (0.883, 1.477) |
|  |  | control (242) | 69 | 123 | 50 |  |  |  |
| rs7903491 |  | | G/G (204) | G/A (536) | A/A (356) |  | | |
|  | **male** | case (317) | 69 | 155 | 93 | 0.517 | 0.083 | 0.814 (0.645, 1.027) |
|  |  | control (308) | 57 | 151 | 100 |  |  |  |
|  | **female** | case (229) | 43 | 112 | 74 | 0.368 | 0.171 | 1.199 (0.925, 1.555) |
|  |  | control (242) | 35 | 118 | 89 |  |  |  |

**Table S2**

| SNP age | | | genotype | | | *P*（genotype） | *P*（Alleles） | OR(95%CI) (Alleles) |
| --- | --- | --- | --- | --- | --- | --- | --- | --- |
|  |  |  |  |  |  |  |  |  |
| rs10937625 |  | | T/T (735) | C/T (329) | C/C (32) |  | | |
|  | **age＜50** | case (98) | 65 | 30 | 3 | 0.99 | 0.993 | 0.998 (0.613, 1.624) |
|  |  | control (120) | 80 | 36 | 4 |  |  |  |
|  | **age≥50** | case (448) | 300 | 135 | 13 | 0.987 | 0.872 | 0.980 (0.768, 1.252) |
|  |  | control (430) | 290 | 128 | 12 |  |  |  |
| rs17590046 |  | | T/T (893) | C/T (199) | C/C (4) |  | | |
|  | **age＜50** | case (98) | 79 | 19 | 0 | 0.863 | 0.851 | 0.940 (0.493, 1.792) |
|  |  | control (120) | 98 | 22 | 0 |  |  |  |
|  | **age≥50** | case (448) | 365 | 81 | 2 | 0.997 | 0.961 | 0.992 (0.721, 1.366) |
|  |  | control (430) | 351 | 77 | 2 |  |  |  |
| rs12764057 |  | | T/T (487) | G/T (509) | G/G (100) |  | | |
|  | **age＜50** | case (98) | 39 | 45 | 4 | 0.425 | 0.605 | 1.117 (0.734, 1.701) |
|  |  | control (120) | 53 | 56 | 11 |  |  |  |
|  | **age≥50** | case (448) | 204 | 208 | 46 | 0.87 | 0.848 | 0.981 (0.804, 1.196) |
|  |  | control (430) | 191 | 200 | 39 |  |  |  |
| rs10822974 |  | | G/G (352) | G/A (560) | A/A (184) |  | | |
|  | **age＜50** | case (98) | 32 | 50 | 16 | 0.998 | 0.959 | 1.010 (0.689, 1.481) |
|  |  | control (120) | 39 | 61 | 20 |  |  |  |
|  | **age≥50** | case (448) | 143 | 229 | 76 | 0.996 | 0.934 | 0.992 (0.821, 1.199) |
|  |  | control (430) | 138 | 220 | 72 |  |  |  |
| rs7903491 |  | | G/G (204) | G/A (536) | A/A (356) |  | | |
|  | **age＜50** | case (98) | 15 | 38 | 45 | 0.196 | 0.161 | 0.757 ( 0.512, 1.118) |
|  |  | control (120) | 20 | 59 | 41 |  |  |  |
|  | **age≥50** | case (448) | 88 | 229 | 131 | 0.612 | 0.412 | 1.082 (0.896, 1.306) |
|  |  | control (430) | 81 | 210 | 139 |  |  |  |

**Table S3**
